# Do Non-Genomically Encoded Fusion Transcripts Cause Recurrent Chromosomal Translocations?

**DOI:** 10.3390/cancers4041036

**Published:** 2012-10-18

**Authors:** Eric Kowarz, Theo Dingermann, Rolf Marschalek

**Affiliations:** Institute of Pharmaceutical Biology/ZAFES/DCAL, Goethe-University of Frankfurt, Biocenter, Max-von-Laue-Str. 9, D-60438 Frankfurt/Main, Germany; E-Mails: eric.kowarz@gmail.com (E.K.); dingermann@em.uni-frankfurt.de (T.D.)

**Keywords:** recurrent chromosomal translocations, leukemia, solid tumors, genetic rearrangements, RNA-templated DNA repair, premature transcript termination

## Abstract

We among others have recently demonstrated that normal cells produce “fusion mRNAs”. These fusion mRNAs do not derive from rearranged genomic loci, but rather they are derived from “early-terminated transcripts” (ETTs). Premature transcriptional termination takes place in intronic sequences that belong to “breakpoint cluster regions”. One important property of ETTs is that they exhibit an unsaturated splice donor site. This results in: (1) splicing to “cryptic exons” present in the final intron; (2) Splicing to another transcript of the same gene (intragenic trans-splicing), resulting in “exon repetitions”; (3) splicing to a transcript of another gene (intergenic trans-splicing), leading to “non-genomically encoded fusion transcripts” (NGEFTs). These NGEFTs bear the potential risk to influence DNA repair processes, since they share identical nucleotides with their DNA of origin, and thus, could be used as “guidance RNA” for DNA repair processes. Here, we present experimental data about four other genes. Three of them are associated with hemato-malignancies (*ETV6*, *NUP98* and *RUNX1*), while one is associated with solid tumors (*EWSR1*). Our results demonstrate that all genes investigated so far (*MLL*, *AF4*, *AF9*, *ENL*, *ELL*, *ETV6*, *NUP98*, *RUNX1* and *EWSR1*) display ETTs and produce transpliced mRNA species, indicating that this is a genuine property of translocating genes.

## 1. Introduction

One of the hallmarks in hemato-malignant disorders and solid tumors are gross chromosomal changes, termed reciprocal (or balanced) chromosomal translocations. These genetic lesions occur by illegitimate recombination events between two non-homologous chromosomes, initiated by a DNA damage situation and the subsequent actions of the non-homologous end joining (NHEJ) DNA repair pathway [1]. The resulting derivative chromosomes encode chimeric genes at their fusion junction. They produce the typical fusion mRNAs that are translated into oncogenic fusion proteins. This initiates a complex process that potentially leads to cancer cells. The presence of these “fusion mRNAs” in cancer cells is assumed to be correlated with the presence of specific chromosomal translocation, and therefore, is widely used as molecular read-out for routine diagnostics.

**Figure 1:**
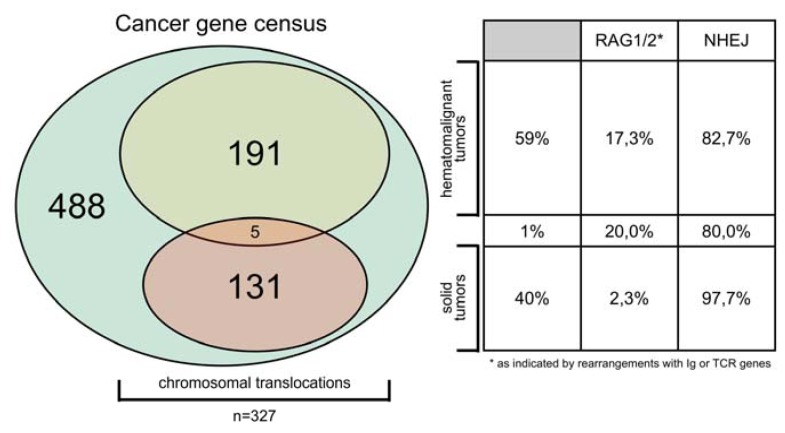
Cancer genes are frequently involved in chromosomal translocations. According to the Cancer gene census, the human genome harbors 488 cancer genes that are recurrently diagnosed in different individuals. Of those, 327 genes are involved in recurrent genomic translocations, leading to the creation of “fusion genes” that encode fusion proteins with oncogenic potential. The group of translocating genes can be separated into 191 genes that are associated with hematomalignant tumors, while 131 are associated with solid tumors. Five genes were associated with both disease entities. Most of these genes are recombined by an NHEJ-mediated recombination mechanism (~83% *vs*. ~98% in hemato-malinant *vs*. solid tumors), while some are recombined by a RAG1/2-mediated mechanism (~17% *vs*. ~2% in hemato-malignant *vs*. solid tumors).

The human genome has about 21,000 genes of which only very few genes are recurrently diagnosed as “mutated” in human tumor samples. So far, the “Cancer gene census” of the Welcome Trust Sanger Institute lists a total of 488 genes, of which 327 are recurrently involved in chromosomal translocations (67%). This group of 327 genes can be subdivided into 191 chromosomal translocations solely associated with the development of hemato-malignant disorders (59%), 131 chromosomal translocations associated with solid tumors (40%), and 5 chromosomal translocations that were identified in hemato-malignant as well as in solid tumors (1%; Figure 1). In summary, only few genes of our genome are recurrently involved in genetic rearrangements and are associated with malignant cell growth. The question arises what kind of property can be attributed to those genes to explain their frequent involvement into illegitimate recombination events.

More than a decade ago, several laboratories identified “fusion mRNAs” in normal cells. For example, *MLL*-partial tandem duplications (PTDs) and *MLL•AF4* fusion transcripts were diagnosed in hematopoietic cells of healthy individuals [2,3]. Fusion transcripts of *BCR•ABL* [4,5], *TEL•AML1* and *AML1•ETO* [6], *PML•RARα* [7], *NPM•ALK* and *ATIC•ALK* [8,9] have been identified in individuals without any genomic rearrangement of the corresponding gene loci. The first interpretation of the authors was that a fraction, presumably a single cell, of the investigated samples bears such a genetic rearrangement. However, all subsequent analyses that were performed to validate this hypothesis by independent techniques (cytogenetic analyses [3,9], genomic PCR [2,8], FISH [7], Southern Blot [2,8]) failed to demonstrate the presence of genetic rearrangements in a convincing manner. This led to the opinion that these studies probably amplified contaminating transcripts, since most of these laboratories were also performing routine diagnostic analyses for exactly those fusion transcripts [9,10].

However, this was not the case, because we demonstrated more than a decade later that normal cells are indeed capable of producing such transcripts. In fact, these fusion transcripts were not produced by genomic alterations rather they are derived from trans-splicing events [11]. cDNA-synthesized from nucleic acids isolated from peripheral mononuclear cells (PBMCs) of healthy individuals demonstrated in nested PCR experiments that e.g., *MLL-AF4* and *NPM-ALK* fusion transcripts can be readily identified in each of the investigated samples. Since we established the *MLL* gene structure [12], and have investigated nearly every aspect of this gene in great detail, we also demonstrated that about 20% of all *MLL* transcripts terminate in *MLL* introns 8 or 9 (*MLL* breakpoint cluster region located between exons 8 and 14). In addition, a cryptic promoter near *MLL* exon 12 allows re-initiation of RNA transcription to express solely the C-terminal portion of the MLL protein [13]. These findings led to additional studies, where we systematically investigated a series of *MLL* fusion partner genes (*AF4*, *AF9*, *ELL* and *ENL*) to demonstrate again that all these genes display a similar feature, namely to produce ETTs in their corresponding breakpoint cluster regions. Inverse RT-PCR experiments revealed the different exon repetitions, and subsequent 3'-RACE experiments uncovered the spectrum of trans-spliced fusion mRNAs for all investigated genes [11].

Of interest, experiments performed by others demonstrated that a *JAZF1-JJAZ1* fusion RNA—normally created by the (7;17)(p15;q21) chromosomal translocation in endometrial stromal sarcomas—can be readily detected in normal endometrial stromal cells [14,15]. Moreover, in normal endometrial cells this non-genomically encoded *JAZF1-JJAZ1* fusion mRNA produces a chimeric protein that exhibits anti-apoptotic effects. Therefore, this trans-spliced fusion mRNAs seems to be made on purpose to gain a survival function.

Assuming that the production of non-genomically encoded fusion transcripts (NGEFTs) is a physiological event that lead to novel cellular functions by using an evolutionary process (exon shuffling), we also posed the question whether the production of NGEFTs in healthy cells may lead under certain circumstances to the generation of chromosomal translocation. If so, it would perfectly explain why certain genes of our genome are recurrently involved in genomic rearrangements.

**Figure 2:**
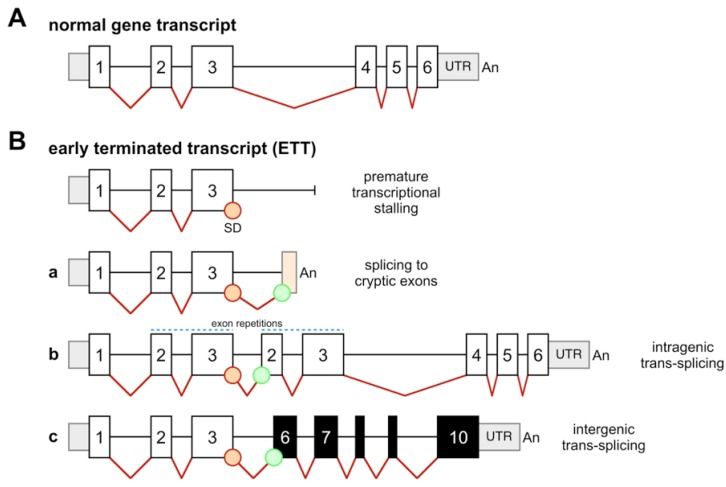
ETTs cause 3 different splice events. (**A**) Genes normally produce a primary transcript that is subsequently spliced into the mature RNA; (**B**) The production of early-terminated transcripts (ETTs) causes three different and independent scenarios. Due to an unsaturated 3'-splicie donor site and based on the available sequences within a given gene, an ETT may cause splicing to a cryptic exon that is encoded by intronic sequences (**a**); In all cases investigated so far, intragenic trans-splicing reactions are causing “exon repetitions” (**b**); This can be explained by the fact that the unsaturated splice donor site is using a splice acceptor site of a second, nascent transcript of the same gene. If genes are co-transcribed in the same transcription factory, then intergenic trans-splicing reactions can be observed which will result in the production of a “non-genomically encoded fusion transcript” (NGEFT) (**c**); Here, the unsaturated splice donor site of the ETT is using a splice acceptor site of a nascent transcript of a second gene. White boxes: exons; lines: introns; grey boxes: 5'- and 3'-UTR sequences; light orange box: cryptic exon. Splice donor (SD) sites are marked as orange circles, while splice acceptor sites are marked as green circles.

Early terminated transcripts (ETTs) exhibit *per se* a unique feature because of their “unsaturated” splice donor sites in the final exon of the prematurely terminated molecule. Such transcripts cause three different scenarios that can be used as experimental read-out: (1) splicing to “cryptic exons” located in the 3' intronic region (Figure 2Ba); (2) intragenic trans-splicing events, leading to transcripts that contain an “exon repetition” (Figure 2Bb); (3) intergenic trans-splicing reactions, causing the production of “non-genomically encoded fusion transcripts” (NGEFTs; Figure 2Bc). While transcripts that contain exon repetitions can be easily identified in RT-PCR experiments, NGEFTs are usually identified by nested PCR experiments. This indicates that intragenic trans-splicing is a much more frequent process than intergenic trans-splicing. This is in line with the concept that transcription takes place in “transcription factories”, where chromatin loops of different chromosomes are transcribed together. If chromatin loops have to enter a transcription factory, then transcription has to be considered as an oscillating process. As a consequence, the probability for co-transcription of two independent genes is much lower than the transcriptional process of a single gene alone. Thus, exon repetitions ought to be more frequently produced from ETTs than intergenic trans-spliced fusion transcripts.

The presence of NGEFTs may cause problems under specific circumstances, namely if a gene—producing such an NGEFT—encounters a DNA damage situation. Upon DNA breakage, a fusion transcript might be used as a “guidance molecule” during the subsequent DNA repair process. A good example for this scenario is the human *MLL* gene. DNA double-strand breaks occur frequently in the breakpoint cluster region that is flanked by the *MLL* exons 8 and 14. The high incidence for DNA breakage events was experimentally attributed to specific chromatin features, e.g., SAR/MAR structures [16,17,18], a DNase I hypersensitive site [19], several Topoisomerase II binding sites [20,21,22,23], a gene internal transcription initiation site [13] and a site-specific endonucleolytical cleavage that occurs early in apoptosis [24,25,26,27]. All these features seem to define a “genetic hot spot” in *MLL*. Noteworthy, we have characterized so far more than 100 gene fusions to the human *MLL* gene [28], indicating that “genetic instability” represents a prerequisite for genomic rearrangements.

Here, we have extended our previous investigations to other genes that are frequently involved in genomic rearrangements. For this purpose we investigated the following genes: *ETV6*, *EWSR1*, *NUP98* and *RUNX1*. We were able to confirm that also these genes produce ETT’s, terminating in their corresponding breakpoint cluster regions, and confirmed ETT production as genuine property of genes involved in recurrent genetic recombination events.

## 2. Results and Discussion

### 2.1. Choosing Additional Genes for Determining EETs and NGEFTs

As summarized in Figure 1, the cancer gene census database (as dated from 15 March 2012) contains a total of 488 human cancer genes, of which 327 genes have been identified in chromosomal translocations. Of those 136 were identified in solid tumors and 196 were identified in hemato-malignant disorders (five genes of both groups were identified in both disease entities: *CCND1*, *FUS*, *LPP*, *MN1* and *EWSR1*). As judged from rearrangements with either an immunoglobulin or a T-cell receptor gene, we assume that most gene rearrangements are based on an NHEJ-mediated process that normally occurs in the G0/1 phase of the cell cycle. In the case of hemato-malignant disorders these are ~83%, while chromosomal translocations in solid tumors are caused in ~98% by NHEJ. RAG1/2-mediated recombination events are—as expected—more frequent in cells deriving from the hematopoietic system rather than from other tissues. We then investigated public databases for known transcript variants of these genes. This led quickly to *ETV6*, *NUP98*, *RUNX1* and *EWSR1* which we investigated further.

The *ETV6* gene (also known as *TEL* gene) is located on chromosome 12p13. It has a size of 241.77 kb and consists of 8 exons that encode a protein of 452 amino acids. *ETV6* is involved in different chromosomal translocations, like the *ETV6-RUNX1* translocation associated with ALL. Based on the data available from the human Atlas of Genetics and Cytogenetic in Oncology and Hematology (AGCOH; edited by Jean-Loup Huret, INSERM U 935, Genetics, Department Medical Information, University Hospital of Poitiers, F-86021 Poitiers, France), the *ETV6* gene is involved in 28 different chromosomal translocations all associated with leukemia. In particular, the *ETV6* gene is fused to *MDS2* (1p36), *ARNT* (1q21), *ABL2* (1q25), *MDS1-EVI1* (3q26), *FGFR3* (4p16), *PDGFRA* (4q11), *CHIC2* (4q11), *ACSL6* (5q31), *PDGFRB* (5q33), *FRK* (6q21), *STL* (6q23), *HLXB9* (7q36), *LYN* (8q12), *NCOA2* (8q13), *JAK2* (9p24), *PAX5* (9p1), *SYK* (9q22), *ABL1* (9q34), *GOT1* (10q24), *BAZ2A* (12q24), *CDX2* (13q12), *FLT3* (13q12), *TTL* (13q14), *IGH* (14q32), *NTRK3* (15q25), *PER1* (17p12), *RUNX1* (21q22) and *MN1* (22q11). Transcript data available from public databases revealed that several transcripts with cryptic exons and prematurely terminated transcripts of *ETV6* have been described. Most translocations occur within *ETV6* intron 5 (e.g., the *ETV6-RUNX1* rearrangement), while other recombination events occur less frequently in *ETV6* introns 1–4. Notably, the *ETV6-RUNX1* fusion gene is quite frequent and can be diagnosed in about 25% of all diagnosed pediatric B-cell precursor ALL patients.

The situation for the *EWSR1* gene is quite similar. This gene is located on chromosome 22q12, has a size of 32.51 kb and consists of 17 exons with a coding capacity of 656 amino acids. The *EWSR1* gene is involved in 18 different chromosomal rearrangements which are associated with Myoepithelial tumors (MET), Ewing sarcomas/peripheral neuroectodermal tumors (ES/PNET), Extraskeletal myxoid chondrosarcoma (EMCS), Myxoid liposarcoma (MLS), Rhabdomyosarcoma (RMS), Angiomatoid fibrous histiocytoma (AFH), Clear cell sarcoma of soft parts (CCSSP), Desmoplastic small round cell tumors (DSRCT) and Acute leukemia (AL). In particular, the EWSR1 gene is fused to *PBX1* (1q23; MET), *SP3* (2q31), *CREBBP1* (2q34; CCSSP, AFH), *FEV* (2q26; ES/PNET), *DUX4* (4q35; RMS), POU5F1 (6p21), *ETV1* (7p22; ES/PNET), *NR4A3* (9q22; EMCS), *WT1* (11p13; DSRCT), *FLI1* (11q24; ES/PNET, RMS), *ZNF384* (12p12; AL), *ATF1* (12q13; CCSSP, AFH), *DDIT3* (12q13; MLS), *ETV4* (17q21; ES/PNET), *ZNF444* (19q13, MET), *NFATC2* (20q13; ES/PNET), *ERG* (21q21; ES/PNET, DSRCT) and *PATZ1* (22q12; ES/PNET). Transcript data available from public databases exhibited again that several transcripts with cryptic exons, premature transcripts and gene internal promoter start site of *EWSR1* have been described. Most translocations occur within *EWSR1* introns 7 and 8, less in *EWSR1* introns 6, 9 and 10. The most frequent recombination partner genes are *FLI1* (85%) and *ERG* (10%).

The *NUP98* gene is highly promiscuous with regard to its recombination spectrum. *NUP98* is located on chromosome 11p15, has a size of 122.78 kb, consists of 33 exons and encodes a protein of 1,800 amino acids. The *NUP98* gene is involved in nearly 30 chromosomal translocation that are all associated with hemato-malignant disorders. In particular, the *NUP98* gene is fused to *PRRX1* (1q24), *HOXD11* (2q31), *HOXD13* (2q31), *TOP2B* (3q24), *LNP1* (3q12), *ANKRD28* (3q24), *JQCG* (4q21), *NSD1* (5q35), *C6orf80* (6q24), *HOXA9* (7p15), *HOXA10* (7p15), *HOXA13* (7p15), *HOXA11* (7p15), *WHSCC1L1* (8p11), *PSIP1* (9p22), *PRRX2* (9q34), *ADD3* (10q24), *HHEX* (10q23), *DDX10* (11q22), *JARID1A* (12q13), *HOXC11* (12q13), *HOXC13* (12q13), *PHF23* (17p13), *SETBP1* (18q21), *TOP1* (20q12) and several other loci that had have not been cloned so far. Transcript data available from public databases displayed again that several transcripts display cryptic exons or are prematurely terminated. Like in the *MLL* gene, a gene internal promoter start site has been described. Most translocations occur within *NUP98* introns 12 and 13, less in *NUP98* introns 8, 9, 10, 11 and 14.

Finally, we chose the *RUNX1* gene (also known as *AML1* gene). *RUNX1* is located on chromosome 21q22, has a size of 261.54 kb. Due to alternative promoters, two major transcripts are produced that consist either of 8 or 6 exons, decoding either a protein of 480 or 452 amino acids. The *RUNX1* gene is involved in nearly 39 chromosomal translocation that are all associated either with hemato-malignant or platelet disorders. In particular, the *RUNX1* gene is fused to *YTHDF2* (1p35), *PRMD16* (1p36), *ZNF687* (1q21), *AFF3* (2q11), *MDS-EVI1* (3q26), *FGA7* (4q28), *SH3D19* (4q31), *USP42* (7p22), *FGFR1* (8p11), *RUNX1T1* (8q22), *ZFPM2* (8q23), *TRPS1* (8q24), *MACROD1* (11q13), *ETV6* (12p13), *CPNE8* (12q12), *CBFA2T3* (16q24), *AMP19* (19q13), *UPS25* (21q11) and many others. Transcript data available from public databases showed again that several transcripts contain cryptic exons, are prematurely terminated or start at a gene internal promoter start site of *RUNX1*. Based on the 8-exon gene structure, almost all genetic rearrangements occur within *RUNX1* introns 5 and 6.

### 2.2. Identification of Early-Terminated Transcripts in Breakpoint Cluster Regions of Frequently Rearranging Genes

**Figure 3:**
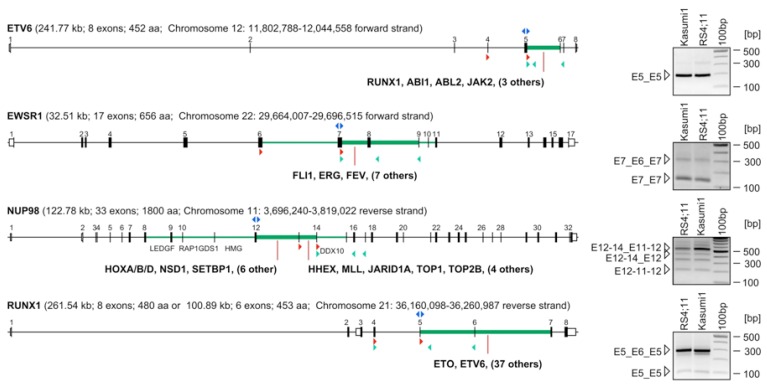
Genes involved in CT display ETTs and exon repetitions. The exon/intron structures of all four investigated genes are displayed: *ETV6*, *EWSR1*, *NUP98* and *RUNX1*. Exons are indicated by black rectangles. The known breakpoint cluster regions of each gene are marked in green; thicker lines indicate major breakpoint cluster regions. Exon numbering is shown on top of each exon/intron structure. 5' and 3'-UTRs are depicted by white rectangles. Genes known to be recombined within a certain intronic region are indicated below. Primers used for exon repetition mapping are indicated above the exon as blue triangles. Oligonucleotides used for 3'-RACE or QRT-PCR experiments are indicated at their corresponding position below the gene structure and depicted as red and green triangles, respectively. Right panels: results of our exon repetition mapping analyses, exemplarily shown for the RS4;11 and Kasumi cell lines. Single bands were sequenced to reveal their exonic composition. Exon repetitions are indicated for each gene on the left of each panel. Sizes are given on the right of each panel.

Based on these data, primers were designed to bind in opposite orientations to the first exon upstream of the breakpoint cluster region for inverse RT-PCR experiments. For the subsequently performed 3'-RACE experiments we always used a nested primer that hybridizes to the major donor exon identified in the corresponding exon repetition experiment. As shown in Figure 3, two primers binding to *ETV6* exon 5 were used to demonstrate the presence of exon repetitions (see right panel). A major exon repetition was identified which fused *ETV6* exon 5 with *ETV6* exon 5. 3'-RACE experiments revealed a series of polyadenylated transcripts that are terminating the *ETV6* transcripts shortly after cryptic poly A-sites within introns 4, 5, 6 and 7. In addition, we identified a cryptic exon within *ETV6* intron 5. We also identified a functional fusion transcript where *ETV6* exon 5 is fused in-frame to *MGST3* exon 6. Significantly, *ETV6-MGST3* fusion transcripts have never been described as chromosomal translocation of the *ETV6* gene (summarized in Table A1 in the Appendix).

Similarly, two oligonucleotides binding to *EWSR1* exon 7 were used to demonstrate again the presence of exon repetitions. Two different exon repetitions were identified, which fused *EWSR1* exon 7 with *EWSR1* exon 7, or, *EWSR1* exon 7 with *EWSR1* exon 6 and 7 (see right panel). In addition, two cryptic exons were identified within *EWSR1* intron 8. 3'-RACE experiments revealed an out-of frame fusion between *EWSR1* exon 7 and *PRPF6* exon 20. A stop codon after 18 codons will lead to premature termination of the fusion protein that is encoded by the first 265 amino acids deriving from the *EWSR1* gene and 18 amino acids deriving from a non-in-frame fused exon 20 of the *PRPF6* gene (summarized in Table A1 in the Appendix). Noteworthy, EWSR1-PRPF6 genomic fusions have never been described as chromosomal translocation in cancer patients, however, the PRPF6 gene is included in the Cancer Census gene database.

For *NUP98* and *RUNX1* no chimeric fusion transcripts could be identified in 3'-RACE experiments. However, both genes displayed exon repetitions, cryptic exons and cryptic poly-A sites (all summarized in Table A1 in the Appendix and Figure 3).

In summary, these data indicate that all four investigated genes—frequently involved in illegitimate recombination events—display a large variety of different early-terminated transcripts. These ETTs take part in trans-splicing reactions that are always cause the production of exon repetitions (intragenic trans-splicing) or trans-spliced fusion mRNA (intergenic trans-splicing).

### 2.3. Abundance of ETTs

Next, we investigated the abundance of these transcripts in all four genes by qRT-PCR. For this purpose we used primers depicted as green triangles in Figure 3. For *ETV6*, *EWSR1* and *RUNX1* one primer is binding to an exon upstream of the premature termination site in conjuction with primers located downstream (intronic or exonic). In the case of *NUP98*, we used only exonic primers because of the unusual localization of the cryptic poly-A site within exon 16. This allowed us to estimate the relative distribution of transcripts that are either full-length or prematurely terminated. To our surprise, the 4 genes produce quite different amounts of ETTS. Relative to the full-length *ETV6* transcript, early termination within intron 5 occurs with a rate of about 15–22%. Early-terminated transcripts within *EWSR1* intron 7 occur only with a frequency of about 5%, while *RUNX1* produces ETTs within intron 5 in about 1%. However, premature termination within *NUP98* exon 16 - bearing a cryptic poly A site—occurs 12–20 times more frequently than longer transcripts are produced. In the case of the *NUP98* gene, only 5–10% of transcripts are extending beyond exon 16. However, these data has to be confirmed in independent experiments using only poly(A)^+^ RNA to exclude any influence of existing pre-mRNAs to be contained in our RNA samples.

## 3. Experimental Section

### 3.1. Cell Lines and Patient Material

For our analyses we used the two t(4;11) cell lines RS4;11 and SEM, as well as the RUNX1-ETV6 cell line Kasumi-1. All three hematopoietic cell lines were cultivated as recommended. PBMCs of healthy individuals were obtained from the Frankfurt Red Cross Blood bank after informed consent of the donors.

### 3.2. Identification of Exon Repetitions and Fusion Transcripts

All RNA samples were extracted from the corresponding cells using the RNeasy-Kit (Qiagen, Hilden, Germany). cDNAs for inverse RT-PCR (RS4;11 and Kasumi-1) or quantitative RT-PCR experiments (PBMCs, n = 3) were reverse transcribed (SuperScript II, Invitrogen, Darmstadt, Germany) from 4–5 µg RNA primed with random hexamers. A dT-anchor primer (GeneRacer-Kit, Invitrogen) was used to generate cDNAs for 3'-RACE experiments (RS4;11 and SEM). PCR-products obtained after inverse RT-PCR or 3'-RACE were separated by gel electrophoresis. The resulting bands were gel extracted (Qiagen) and cloned for sequencing. Quantitative RT-PCR experiments were performed on a StepOnePlus machine (Applied Biosystems, Darmstadt, Germany) using SYBR Green.

The inverse primer sets used for the identification of exon repetitions were: ETV6.E5F (5'-AAGACCTGGCTTACATGAACCACATCATGG-3') and ETV6.E5R (5'-GGTGATTTGTCGTGATAGGTGACCTGGAG-3'); EWSR1.E7F (5'-AAACTGGATCCTACAGCCAAGCT-CCAAGTC-3') and EWSR1.E7R (5'-GGTTGCCCATAGGTGTTCTGCTGAGAGTAA-3'); RUNX1.E5F (5'-CCACAGAGCCATCAAAATCACAGTGGATG-3') and RUNX1.E5R (5'-AC-TTGCGGTGGGTTTGTGAAGACAGTGAT-3'); NUP98.E12F (5'-ATACTACGACAGCCACTT-TGGGCTTTGGAG-3') and NUP98.E12R (5'-CCTGTACCAAGAGGCCCTCCAATCTTAGGT-3').

For 3'-RACE experiments two rounds of PCR were performed using the 3'-GeneRacer nested primers (Invitrogen) and nested gene-specific primers: ETV6.E4F (5-TCACCATTCTTCCACCCTGGAAACTCTAT-3') and ETV6.E5F; EWSR1.E6F (5'-CAGCCCAGCCTAGGATATGGACAGAGTAAC-3') and EWSR1.E7F; NUP98.E13F (5'-CGGAATCCGATGTCAGACCCTAAGAAGAAG-3') and NUP98.E14F (5'-ACTAGAGTCCGGCCAAAGGCTTTACAAACA-3'); RUNX1.E4F (5'-CAGGTTGCAAGATTTAATGACCTCAGGTTT-3') and RUNX1.E5F.

The following primers were used for quantitative RT-PCR experiments: ETV6.E5F, ETV6.I5R (5'-CATTTCTGAGGCTCATTAACTACTCCATCC-3'), ETV6.E7R (5'-GTCCTGGCTCCTTCCTGATAATGTTTAGTT-3'); EWSR1.E7F, EWSR1.RT.I8R (5'-TCTTTCAGTCCCTTCTTTCCACTGA-3'), EWSR1.RT.E9R (5'-CTTATTGAAGCCACCTCGCTCTCCAG-3'); RUNX1.E4F, RUNX1.I5R (5'-AAGGAATCTGAGACATGGTCCCTGAGTAT-3'), RUNX1.E6R (5'-GGTCTG-ATCATCTAGTTTCTGCCGATGT-3'); NUP98.E14F (5'-CTTTACAAACAACAGGCACAGC-CAAGTCAC-3'), NUP98.E16R (5'-GTTTGAGGAATAGGTTTGGCAATAGGGTTA-3'), NUP98.E17R (5'-CTTAGTGAGAATAATACCTGCTGGGTGCAT-3').

## 4. Conclusions

Here, we present additional evidence that extends our previous study where we have investigated in depth the trans-splicing properties of *MLL* and some of its major fusion genes [11]. *MLL*, *AF4*, *AF9*, *ELL* and *ENL* produce early-terminated transcripts, which result in a large variety of trans-spliced RNA species. This could now be demonstrated for four additional genes, *ETV6*, *EWSR1*, *NUP98* and *RUNX1*, respectively. Thus, all genes which have been investigated so far displayed the same molecular feature, namely the production of ETTs. These transcripts always terminate in genomic regions classified as “breakpoint cluster regions”. ETTs exhibit an unsaturated 3'-splice donor site that needs to be saturated. As a result, transcripts exhibiting “exon repetitions” (ETTs) or “non-genomically encoded fusion transcripts” (NGEFTs) are produced. The latter transcripts mimic the presence of genetic alterations, which are not present at the genomic DNA level in these cells. In all cases where we tested for the presence of corresponding reciprocal fusion transcripts we were not able to identify them. This is currently the only possible experimental readout to validate the presence of a given NGEFTs and to concomitantly exclude the presence of a given chromosomal translocation in which a reciprocal fusion transcript should be detectable.

Assuming that our applied PCR techniques select shorter RNA variants, and taking into account that we analyzed only a few cell lines and PBMCs of healthy individuals, we can conclude that we only visualized the tip of the iceberg in our experiments. Based on our data, we assume that NGEFTs are produced in a tissue-specific manner, indicating that certain genes are transcribed together in a common transcription factory. This might be explained by chromosome territories that are localized in vicinity of the 3-dimensional space of the nucleus. Chromatin loops of these chromosome surfaces may protrude into these factories and initiate the hnRNA production. Given the fact that differentiation processes are changing the transcriptional profile of a given cell, and that the total amount of active and inactive genes somehow determines the 3-dimensional shape of the chromosomes, differentiation processes will always change the set of genes that are potentially transcribed together in a single transcription factory. Here, we only used four cell lines and PBMCs from healthy individuals, but we were still able to detect the production of an *ETV6-MGST3* and an *EWSR1*-*PRPF6* trans-spliced fusion RNA.

Since ETTs may easily cause the production of NGEFTs, we propose that the production of trans-spliced fusion transcripts is a genetic prerequisite for genetic rearrangements. However, NGEFT production will only have an effect on genetic integrity if this combines with a high susceptibility for DNA lesions. DNA damage is the important molecular trigger for the onset of genetic rearrangements. In the case of a DNA double strand break, adjacent transcripts may help to sort out how broken chromosome ends should be aligned prior to DNA repair. Therefore, we believe that the presence of RNA molecules in general affects subsequent DNA repair processes. This phenomenon has been termed “RNA-templated DNA repair”, and was successfully demonstrated in the fission yeast [29,30]. We have already depicted a molecular mechanism that could explain how a given NGEFT might lead to chromosomal translocations in higher eukaryotic cells (see the fourth figure in [11]). *Vice versa*, normal hnRNA transcripts may have an important biological function, namely to maintain genetic integrity. Notably, the ENCODE project has already discovered that major parts of the human genome is encoded by hnRNA molecules [31]. Since this represents a novel function of RNA in cell biology, additional studies will be needed to investigate more genes of the cancer gene census database in order to evaluate their ETT producing capacity. This will help to evaluate our findings and to push forward a novel concept in biology, namely the influence of RNA on DNA repair, and in the case of NGEFT, how an erroneous repair could lead to recurrent chromosomal translocation [32,33].

## Appendix

**Table A1:** Results of 3'-RACE experiments. All data concerning the four investigated genes. (**A**) ETV6; (**B**) EWSR1; (**C**) NUP98; (**D**) RUNX1. Data were separated into the identified cryptic exons, cryptic poly A sites and trans-spliced fusion transcripts. Only the non-genomically encoded polyA streches were indicated as (a_n_).

**(A) ETV6—3'RACE experiments**
cryptic exon within intron 5
(tag|)agacggggtttcaccatgttagccaggatggtcttgatctcctgacctcgcgatccgcctgcgtcggcctcctaaagtgctaggattacaggcgtgagccaccgcgcctggccggtactgtcccttttttatttaaatcatgtatttctagatttttctctggccccaagtgcaccaccagtgttgtctcagtttcactgttataaatcatttacatcagggatgttgaatccagaaaataaaatggcaaacaaaaatga(a_n_)
cryptic poly-A site intron 4: …ttcttgcctgaggtttagacaaatccaggaagtttaattgttaacttgataagctggtc(a_n_)
cryptic poly-A site intron 5: …acatcagggatgttgaatccagaaaataaaatggcaaacaaaaa(a_n_)
cryptic poly-A site intron 5: …agcgaaaacatttaaaataaaaggtttatgagcatgc(a_n_)
cryptic poly-A site intron 5: …cattgagcacctactgtctgccagagtaataaagcgctgtagaagcaaagca(a_n_)
cryptic poly-A site intron 6: …gaaggaaataattaaagatgcatcttgccatttgttcctga(a_n_)
cryptic poly-A site intron 7: …ttatgtctgtcctgtaataaagccaaaactaaatgaaca(a_n_)
ETV6 fusion mRNA: ETV6 exon 5::MGST3 exon 6 (in-frame fusion):
CACGCCATGCCCATTGGGAGAATAGCAG::AACCCAGCAAGCGTAGTCGAGGAGCCCT
[microsomal glutathione S-transferase 3 (MGST3) is located on chromosome 1q24, consists of 6 exons and encodes 152 amino acids]
**(B) EWSR1—3'-RACE experiments**
cryptic exon within intron 8
(cag|)gttacaaagtgagagccttgtatacacttcaatacttaaaaagtacccgtactcagtactcagccggcagcataatgaaaagtgggactagacacggtgtccatatggagaggaaaaatatacatagaatatttttaacaaaatgtattcattgtataaatggaatccttctgtaactttggtaactgcatacttgttgtttggtaatgaaccagaggaggtataatactctagaattgtgtaacattaaagtgtaaacttttgtgtttaaa(a_n_)
cryptic exon within intron 8
(cag|)gttacaaagtgagagccttgtatacacttcaatacttaaaaagtacccgtactcagtactcagccggcagcataatgaaaagtgggactagacacggtgtccatatggagaggaaaaatatacatagaatatttttaacaaaatgtattcattgta(a_n_)
EWSR1 fusion mRNA: EWSR1 exon 7::PRPF6 exon 20 (out-of-frame fusion):
CAACAGAGCAGCAGCTACGGGCAGCAGA::GCTGTTTTGGAGTCAGCGGAAGATCACCA
[pre-mRNA processing factor 6 (PRP6) is located on 20q13.3, consists of 21 exons and encodes 941 amino acids]
**(C) NUP98—3'-RACE experiments**
cryptic poly-A site exon 16: TGCTGGAAATAAACACAGCAACAGCAA(a_n_)
additional exon repetition: E14_E13-14-15-16 (out-of-frame; poly-adenylated within exon 16)
**(D) RUNX1—3'-RACE experiments**
cryptic exon within intron 6
(cag|)atggggtttcagcatgttggccaggctcgtctcgaactcctgaccacaggt---gctacaaaaggatttcttttaaccaa(a_n_)
cryptic poly-A site intron 5: ccccgtaaagccaaaaagttgcattgtcaatactctcgctactcaaa(a_n_)
cryptic poly-A site intron 5: tactcagggaccatgtctcagattccttgggttcaac(a_n_)
